# Pure Air in Hospitals

**Published:** 1910-07-16

**Authors:** 


### PURE AIR IN HOSPITALS.

### THE APPLICATION OF OZONE AS USED IN AMERICA.

Some little while back the present writer contributed an article to the pages of The Hospital explaining why, according to his views, the hospital staff usually objected to having the Plenum system in their own quarters, and why it was found that convalescents were sometimes long in making recovery in rooms where the ventilation was on the Plenum system. It will be remembered that the reason he made out to be the smaller quantity of oxygen present in the atmosphere under the conditions ruling with the Plenum system in most hospitals, as against the usual conditions when rooms are left to the action of what is known as natural ventilation. In a subsequent article he suggested that in certain cases ozone, or oxygen if preferred, might be added to the ventilating air current of particular rooms, and especially to that of operating theatres, with a view of maintaining the atmosphere in the operating theatre in such a condition that the exhaustion so frequently felt by surgeons and nurses would be lessened. Since those articles were written he has obtained information from America that something of the kind is actually in practice there in Chicago. The Chicago Public Library appears to have been the first building to which the arrangement was applied. Complaints had been made of the unpleasant smell commonly prevalent in the library, due partly to the mould that is always more or less present where old books are, and partly to the fact that the out-of-works made the library their home.

### The Apparatus Employed.

The apparatus employed, which is made by the National Air Filter Company, of Chicago, is very similar to ozonisers that are made in this country and that have been shown at different exhibitions. An alternating current of electricity at very high tension—8,000 volts and higher—is discharged between plates arranged for the purpose inside a chamber, through which air is driven by the aid of a fan. The high-tension discharge, which is described as violet coloured and silent, oxidises a portion of the oxygen of the air passing through the plates, which are perforated to allow of the passage of the air, ozone being produced. The ozone mixes with the remainder of the air passing through the ozoniser, and the whole of the air current, with the ozone produced, is allowed to pass into the duct of the Plenum ventilating system.

At the Chicago Free Library the air is submitted to the usual treatment common with the Plenum system. It is washed, cooled in summer, warmed in winter, and deprived of all foreign matter. The ozoniser is made to discharge into the same duct that carries the air from the cleaning apparatus into the library, and thence, after passing through the different rooms, to the outlet ducts in the usual way. It is claimed that all living organisms are destroyed by the high-tension current in passing through the ozoniser, and that the ozone which passes into the building effectually purifies the air there, carrying off the unpleasant odours that previously existed.

A writer in an American journal who visited the reading-room of the library states that "the room no longer smelt like a cheap lodging-house, but the air approximated to the breeziness of the hurricane deck of a liner in mid-ocean." It will be remembered that the good effects of sea air are largely due to the ozone that is present, particularly in mid-ocean, and its oxidising effects hardly need enlarging upon.

The writer suggests that a similar apparatus, arranged to discharge into the ducts leading to operating theatres, and controlled from the theatre itself by electrical or other arrangement, would enable the atmosphere of the theatre to be kept more in that breezy state that is so conducive to good work of every kind.

In Chicago and in other American cities, where the apparatus has been applied, it is arranged to run the whole affair from the electric-light service. The "Air Filter," as the makers have termed it, the ozoniser as it would more properly be called, is made in several sizes for use in rooms containing different volumes of air. One form is arranged to be placed in a bedroom, another of a size suitable for halls, restaurants, theatres, etc. For the Chicago Free Library a specially large one was designed, stated to pass 1,200,000 cubic feet of ozonised air through the reading-room every hour, the whole of the air being renewed five times an hour. The apparatus can be put in operation by means of a flexible cord and wall plug, just as a portable electric lamp can. If preferred, the ozoniser could be placed in the operating theatre itself, and would be more completely under control. The National Air Filter Company, of Chicago, the makers of the apparatus, state that they are fitting it in several hospitals in America, and also in the ventilating plant of the Chicago Auditorium Theatre, which is stated to have the largest seating capacity of any theatre in America. The ozoniser is made to discharge into the duct of the ventilating air current of the theatre, just as in the case of the Chicago Free Library, the volume of air ozonised in this case being 150,000 cubic feet per minute. The air filter has been applied in America to the purification of the air of kitchens, of lavatories, of sick rooms, and pretty well every place where pure air is of advantage.

